# Isolated Pancreatic Metastases of Renal Cell Cancer: Genetics and Epigenetics of an Unusual Tumour Entity

**DOI:** 10.3390/cancers14061539

**Published:** 2022-03-17

**Authors:** Franz Sellner, Sabine Thalhammer, Martin Klimpfinger

**Affiliations:** 1 Department of General-, Visceral- and Vascular Surgery, Clinic Favoriten—Kaiser Franz Josef Hospital, 1100 Vienna, Austria; sabine.thalhammer@gesundheitsverbund.at; 2 Clinical Institute of Pathology, Medical University, 1090 Vienna, Austria; martin.klimpfinger@meduniwien.ac.at

**Keywords:** renal cell carcinoma, isolated pancreatic metastasis, seed and soil mechanism, organotropism, prognosis

## Abstract

**Simple Summary:**

The entity of isolated pancreatic metastases is very rare in metastatic renal cell carcinoma and is characterized by unusual features: 1. The isolated occurrence of pancreatic metastases itself; 2. the long interval between treatment of renal cell carcinoma and the occurrence of pancreatic metastases (9.6 years); 3. the frequent multiple occurrence of isolated pancreatic metastases (36%); and 4. the favourable prognosis after surgical therapy (5-year survival rate 75%). Some of the causes of this protracted course can already be traced back to specific genetic/epigenetic changes.

**Abstract:**

Isolated pancreatic metastases of renal cell carcinoma (isPMRCC) are a rare manifestation of metastatic renal cell carcinoma (mRCC) characterized by two peculiarities: (1). The definite or at least long-term exclusive occurrence of metastases in the pancreas and (2). an unusual low tumour aggressiveness with slow tumour progression and consecutive, good treatment results. According to current knowledge, the exclusive occurrence of pancreatic metastases is due to a highly specific and highly selective seed and soil mechanism, which does not allow metastases settlement outside the pancreas, and whose detailed genetic/epigenetic causes are not yet elucidated. Recent studies have shed light on some of the pathways involved for the protracted course of the disease and highlighted a special genetic profile (lack of loss of 9p, lower weight genome instability index, low frequency of BAP1 alterations, and a high frequency of PBRM1 loss), which deviates from the conventional mRCC profile. Finally, the question of the reasons for the long-term relative genetic stability of the involved cell clones, which is an essential prerequisite for a favourable prognosis, remains unanswered.

## 1. Introduction

In rare cases, the pancreas may be the site of distant metastases of extrapancreatic primary tumours. These tumours include, in particular, clear cell renal cell carcinomas, lung carcinomas, colorectal carcinomas, melanomas, and sarcomas [1,2,3,4,5,6,7]. Clear cell renal carcinoma is by far the most common primary tumour, accounting for 60% of pancreatic metastases (PM) [1,2,3,4,7,8,9,10,11,12,13,14,15]. In this review, the isolated pancreatic metastases of renal cell carcinoma (isPMRCC) are presented. In this rare entity, the pancreas itself becomes the exclusive site of distant metastases of the renal cell carcinoma (RCC). In general, pancreatic metastases that rarely occur in terminal systemic disease of tumours (~2% in surgical series; 1.6–11% in autopsy studies [2,5,7,16,17]) have a correspondingly poor prognosis [2,18,19]. In contrast, however, the entity of isPMRCC is characterized by an unexpectedly slow progress and better prognosis [15].

The aim of the review is therefore to present the particularities of isPMRCC by means of a systematic literature review with meta-analysis and, as far as is currently known, to show the genetic and epigenetic mechanisms that might explain this unexpected course.

## 2. Materials and Methods

The repeatedly presented MEDLINE (PubMed)-based database on isPMRCC (Key words: renal cell cancer & pancreatic metastasis) [20,21,22] was updated and now includes 1433 isPMRCC observations from the first description 1950 [23] to the end of 2021 [1,2,3,4,6,7,8,9,10,11,12,18,19,23,24,25,26,27,28,29,30,31,32,33,34,35,36,37,38,39,40,41,42,43,44,45,46,47,48,49,50,51,52,53,54,55,56,57,58,59,60,61,62,63,64,65,66,67,68,69,70,71,72,73,74,75,76,77,78,79,80,81,82,83,84,85,86,87,88,89,90,91,92,93,94,95,96,97,98,99,100,101,102,103,104,105,106,107,108,109,110,111,112,113,114,115,116,117,118,119,120,121,122,123,124,125,126,127,128,129,130,131,132,133,134,135,136,137,138,139,140,141,142,143,144,145,146,147,148,149,150,151,152,153,154,155,156,157,158,159,160,161,162,163,164,165,166,167,168,169,170,171,172,173,174,175,176,177,178,179,180,181,182,183,184,185,186,187,188,189,190,191,192,193,194,195,196,197,198,199,200,201,202,203,204,205,206,207,208,209,210,211,212,213,214,215,216,217,218,219,220,221,222,223,224,225,226,227,228,229,230,231,232,233,234,235,236,237,238,239,240,241,242,243,244,245,246,247,248,249,250,251,252,253].

**Figure 1:**
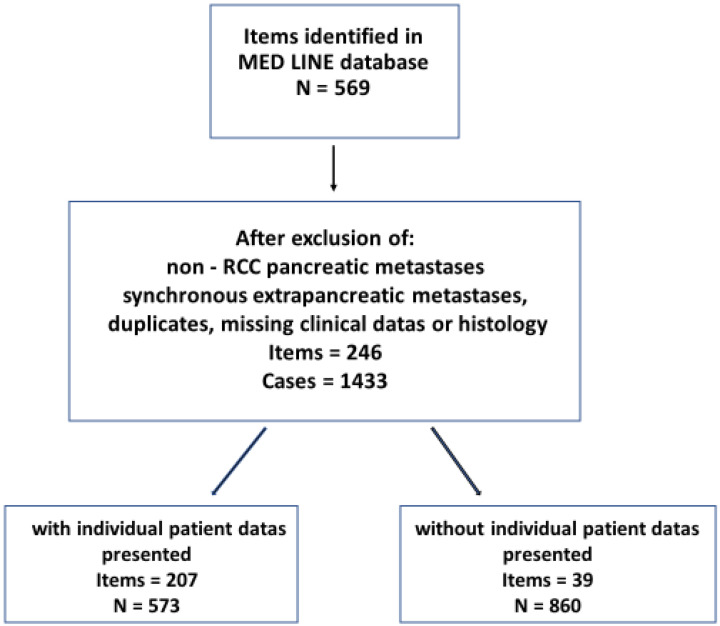
Search and selection strategy.

Inclusion criteria for the database were reports of isPMRCC, i.e., synchronous or metachronous occurring pancreatic metastases (PM) of renal cell carcinoma without metastases in other organs at the time of PM diagnosis, or within 6 months before or after isPM diagnosis. Observations were excluded: (1). If the results did not clearly differentiate PM from RCC and PM from other (non-renal) primary tumours; (2). if cases with additional (oligometastatic) extrapancreatic distant metastases were included in the collective of isPMRCC; and 3. if histology or detailed clinical data on the isPMRCC observations were missing. These observations were separated into those that presented observations in casuistic form and those that reported the summarized results of single institution or multicentre analyses (Figure 1).

For calculations of the influence of PM number, only those reports that cited an exact number or reported unquestionably on single or multiple PM were analysed. An analogous procedure was applied to synchronous or metachronous metastases. To determine the localization of the metastases within the pancreas, only those isPMRCC that explicitly indicated the localization in the head, body, or tail region were used. In the few cases of one institution repeatedly reporting their isPMRCC observations (e.g., (Dept. of Surgery University of Heidelberg; Johns Hopkins University School of Medicine; Verona University of Verona Hospital Trust)), the most detailed report was selected for analysis.

### Statistics

Continuous data is presented as mean (standard deviation). Differences were calculated with Fisher’s exact test (categorial variables) and non-parametric binominal distribution test (dichotomous variables). Survival was calculated according to the Kaplan–Meier method and differences among subgroups were compared by log-rank test. The level of statistical significance was set to <0.05.

## 3. Results

### 3.1. The Clinical Presentation of isPMRCC

In addition to the exclusive occurrence of metastases in the pancreas, isPMRCC are characterized by: (1). a long interval between RCC treatment and manifestation of isPMRCC, (2). the frequent occurrence of multiple metastases in the pancreas, and (3). the favourable treatment results (Table 1).

### 3.2. Interval

In only 7.4% of cases does the isPMRCC occur synchronously with the RCC. In the majority (92.6%), a metachronous occurrence was observed. In those metachronous observations, a long interval from RCC therapy to the manifestation of PM is a characteristic of isPMRCC. A mean interval of 9.6 years (SD 6.5) is calculated from 855 observations. In single and multi-centre reports [7,8,9,11,18,100,104,118,121,124,137,138,152,158,159,179,189,194,195,197,198,204,207,208,213,232,239,240,242,243,246,248,250] values of 5–14 years are reported, with a median of 8.4 years. The longest observed interval is 36 years [235]. The isPMRCC thus undoubtedly counts to the RCC observations with a prolonged clinical course occurring in 15–25% of cases, with periods of slow tumour growth or even stability for many years [152,171,194,206,254,255].

### 3.3. Solitary/Multiple Occurrence

A frequency of solitary PM of 63.6% can be calculated from 733 observations, whereas multiple PM were observed in 36.4%—a surprisingly high incidence in the pancreas measuring only 80 mL [256]. The largest reported number of metastases was 15 foci [18], the mean value of multiple PM was 3.1 metastases and occasionally a diffuse infiltration of the pancreas was described [114]. Large institutional reports (N > 20) [8,11,18,100,104,195,198,238,241,247] calculated the frequency of multiple PM with 25% [11] to 62% [238], with a median of 40%.

## 4. Metastatic Pathway and Seed and Soil Mechanism in isPMRCC

In early publications, it was suggested that the topographical proximity of the ren and pancreas is responsible for isPMRCC via direct lymphatic anastomoses after blockade of regional lymph nodes (LN) [44,88,101,106,159,173,178,191]; by acquired, pathological tumour vessels of hypervascularized tumours [32,44,88,101,106,136,159,172,178,189]; or by pre-existing renal-portal anastomoses [106,257,258]. The importance of this local MP was called into question only by the rapidly increasing number of cases in recent decades, which allowed for epidemiological meta-analyses, and pointed out the importance of a hematogenous systemic MP with subsequent “Seed and Soil Mechanism” (SSM) [21]. Since these arguments were previously presented in detail [16,22], they are summarized here in abbreviated form.

(a) The independence of the distribution of metastases within the pancreas from the site of the RCC in the right or left kidney (Table 2). This behaviour was confirmed in extensive compilations and large single institution reports, too [3,11,13,16,22,197,198,202].
(b) The distribution of metastases within the pancreas (head N = 141; 48.1%; body and tail N = 152; 51.9%) corresponds to the volume proportions of caput (46%) versus corpus and cauda (54%) [259] and does not indicate any preference for a pancreatic part (*p* = 0.206).
(c) In the few observations in which RCC metastases had to be removed before or that occurred after isPMRCC treatment, there was a clear dominance of systemic hematogenic metastases. Of the metastases that had to be removed between RCC and isPMRCC therapy, 78.3% (N = 36) were undoubted systemic hematogenic metastases [4,9,12,18,44,77,84,85,88,103,121,145,152,155,156,174,180,201,202,206,236]. Of the 125 observations in which further metachronous metastases had to be removed after isPMRCC removal, 74.4% were undoubtedly hematogenic distant metastases [4,9,18,20,43,85,88,90,100,101,102,105,106,110,112,124,128,134,139,142,144,152,155,156,157,164,173,174,179,181,190,194,217,229].
(d) The rate of regional peripancreatic lymph node metastases is low in this entity. According to literature data, it is only 6.2% [11,124,179,193,195,197,200,202,230,238,241,247].
(e) The rate of subsequent liver metastases is also not increased in isPMRCC. Literature data shows a value of only 8.0% [9,16,18,32,86,90,101,102,106,139,155,156,173,174,194,226]. While the points (a)–(c) can only be explained by an high importance of a systemic hematogenic MP, the points (d) and (e) confirm the low importance of local—lymphatic or venous—MP.

However, if one is willing to accept the high impact of a systemic MP in this entity as derived from epidemiological studies, the question inevitably arises: Why do metastases occur only and exclusively in the pancreas in spite of systemic tumour cell spread? This is even more true if one takes into account the multiple pancreatic metastases observed in around 36%, which are undoubtedly caused by multiple cell embolisms. That all these cell embolisms by chance only reach the pancreas with a size of only 80 mL [256] is absolutely implausible.

As early as 1889, Paget [260] showed for the first time that the distribution of metastases is not random, but that the individual tumour entities have preferred metastasis host organs. He aptly described this behaviour as “seed and soil” mechanism, because successful metastasis requires a successful multi-stage, cascade-like interaction of many complex properties of the tumour cells (seed) with equally diverse properties of the host organ (soil). Metastasis settlement is therefore only possible if these properties match each other exactly. Already the disturbance of one step in this complex process can interrupt the formation of metastases [261,262] Although all genetic and epigenetic preconditions involved in the early metastasis process are of course not yet known in detail, research over the past decades has already defined the main steps [261,263] that must take place from the arrival of the embolized tumour cell to the clinically manifest metastasis (Table 3)

In summary, these absolutely necessary interactions between the embolized tumour cells and the host organ result in a selection process in metastasis settlement, whereby the different organ tumours have different predilection organs for metastases (e.g., prostate cancer and bone metastases). Such a selective metastasis mechanism must also be suspected for isPMRCC [21], however, with the unusual peculiarity that the metastases do not occur somewhat more frequently in the pancreas in addition to other distant organ metastases, but that there is an absolute selection. Due to as yet unknown mechanisms, the embolized tumour cells can only and exclusively grow to metastases in the pancreas, while in all other organs they undergo cell death/apoptosis.

## 5. Treatment

### 5.1. Surgical Treatment

**Figure 2:**
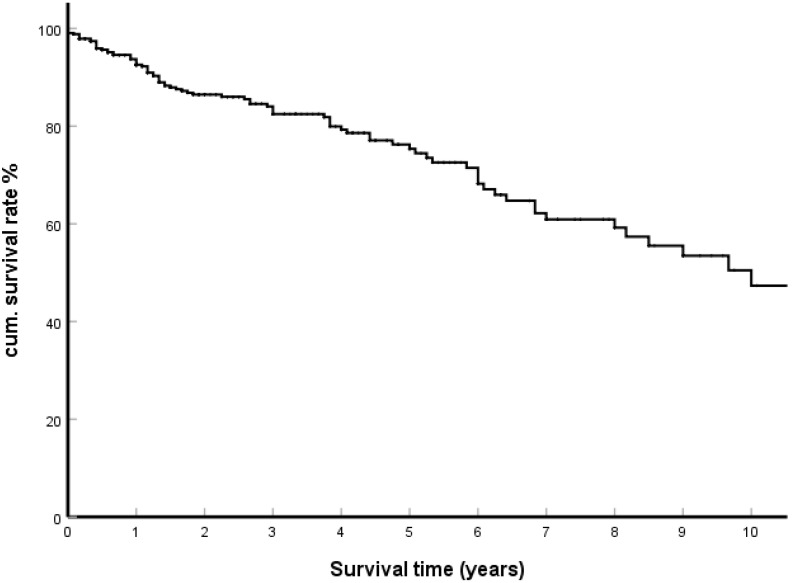
Kaplan–Meier survival curves; surgical treatment group (N = 415).

For many years, surgical removal was the only effective therapy for isPMRCC. Depending on the location and number of metastases within the pancreas, this can be performed as partial duodenopancreatectomy, distal pancreatectomy, total DP, central pancreatectomy, and local tumour resection. In general, the surgical procedures provide a surprisingly good result for metastasis surgery. From 415 casuistically reported observations, a cumulative 5-year and 10-year survival rate of 75.7% and 47.3% respectively was calculated (Figure 2).

These values are consistent with the results of single and multicentre reports. For the median OS [6,11,18,106,152,159,174,179,189,197,208,213,238,241,246], values range from 48 to 134 months, with a median of 75 months. The reported 5-year survival rates are: (Thompson [45] 43%, Madkhali [82] 50%, Tosoian [57] 52%, Bassi [54] 53%, Wente [38] 53%, Wiltberger [8] 56%, Konstantinidis [47] 61%, Schwarz [51] 63%, You [30] 66%, Bauschke [246] 68%, Ito [5] 69%, Chatzizacharias [32] 71%, Fikatas [53] 71%, Di Franco [33] 72%, Benhaim [55] 72%, Law [35] 75%, Kimura [44] 77%, Yuasa [39] 79%, Milanetto [36] 80%, Crippa [31] 80%, Zhang [59] 81%; Blanco-Fernandez [27] 83%, Zerbi [71] 88%, Chikhladze [7] 89%, and Bahra [148] 100% [2,3,11,18,19,104,121,124,137,138,148,155,174,189,195,197,198,204,208,213,238,241,245,246,247], which range from 43% [104] to 100% [148] with a median of 71% [213]; 10-year survival rates are 32% (Schwarz [195]) and 24% (Thompson [104]), respectively.

### 5.2. Recurrence

Following isPMRCC therapy, tumour recurrence with metastases occurred in 125 of 301 sufficiently documented casuistic observations (41.5%) after an interval of 30 months (SD 25 months). The longest reported interval to tumour recurrence—it was a resectable recurrence in the lung—was 10 years [124]. These metastases occurred in 87 of 125 (69.6%) in only one organ. Of these, 19 (21.8%) were again located only in the pancreas [10,101,124,155,179,188,192,194,196,199,211,243] and 9 (10.3%) in an endocrine organ [20,43,51,100,112,134,156,193,194].

Institutional studies (Table 4) report similar results [4,8,9,18,124,174,179,195,198,207,208,213,232,238,239,241,247,249]: After a median interval of 27 months (14–40 months), a median recurrence rate of 44% (0–80%) was observed, of which 92% [247], 70% [195], and 61% [249] were single organ metastases and 24% (9–62% [31,51]) were again pancreatic metastases.

The observation that 10.3% of the single organ relapses after isPMRCC therapy occurred in endocrine organs is noteworthy, since endocrine organs only have about 2% of the volume of the large metastatic organs (liver, lung, bone marrow, and brain) [22]. This may indicate a selection mechanism towards endocrine organs. In this connection it should be noted that the pancreas consists of two different components, an exocrine and an endocrine one, and it is currently unknown whether the isPMRCC originates in the exocrine or endocrine tissue or in both. The possibility that the isPMRCC represent only a special form of affinity of some RCC tumour cells to endocrine tissues is thus at least conceivable.

### 5.3. Systemic Treatment

In recent decades, a highly effective drug therapy for metastatic RCC (mRCC) was established in the form of targeted therapy with tyrosine kinase inhibitors (TKI); mTor inhibitors; and immune checkpoint inhibitors such as anti-PD1, anti-PD L1, and anti-CTLA4 [264,265,266,267,268,269,270,271,272,273], which revolutionized the treatment of patients with advanced RCC [274,275]. These therapies led to favourable results, which were also confirmed in the isPMRCC [176,190,217,276,277,278]. In a large-scale multicentre study, TKI in patients with isPMRCC identified results that could be measured with the surgical results [203]. In immunotherapy (IT), on the other hand, the study by Singla [244]—the only one so far to analyse a significant number of PM in mRCC—showed that it remains ineffective in mRCC with pancreatic metastases, both in isolated PM and in PM in multi-organ metastasis.

## 6. Significance of Risk Factors

Research over the past decades has shown that risk factors associated with tumour volume and tumour growth are also of prognostic importance with advanced mRCC [279,280,281], such as the number of metastases, the number of metastatic sites, and interval from renal tumour to metastases.

It is therefore all the more remarkable that these risk factors do not have a prognostic significance in the isPMRCC. The increasing number of case studies (N = 573) but also newer voluminous single institution and multicentre reports revealed the ineffectiveness of established clinical risk factors that reflect tumour volume and tumour growth rate [16]. These risk factors, which are ultimately a measure of the risk of later tumour progression from occult micrometastases after radical surgery, include singular/multiple metastases, size of singular metastases, number of metastases, synchronous/metachronous occurrence, and duration of interval to occurrence of PM [22]. This phenomenon was already identified in 2006 [20] and has since been confirmed in at least nine major (N > 20) surgical reports [8,11,13,195,197,198,238,241,249]) and four literature reviews [12,58,164,207], as has already been shown [22].

In summary, the clinical course of isPMRCC, both by the long interval to the occurrence of PM and even more by the favourable treatment results, suggests that isPMRCC is composed of cell clones characterized by a low aggressiveness, which is maintained for many years.

## 7. PM and OS of mRCC

The occurrence of PM allows the identification and comparison of three courses in mRCC: mRCC without PM, mRCC with PM, and observations with isolated PM (Table 5). An analysis shows that in all eight studies presented so far, the median OS in multiple organ sites metastases with simultaneous PM were significantly better than for observations without PM (41.7 vs. 23 months) [204,244,282,283,284,285,286,287]. The fact that the reported results for isolated PM are even distinctly better (75 months) may at first glance be attributed to a generally more favourable course of single organ metastases. However, a comparison with other single organ metastases of the RCC, e.g., isolated lung metastases, shows that the median OS in isolated PM at 75 months is 30% better than in isolated lung metastases (56 months). In summary, it should be noted that PM in mRCC—both for isolated PM and PM in the context of multiple organ site metastases—obviously signals a more favourable course in mRCC.

## 8. Discussion

The clinical course of isPMRCC is characterized by two peculiarities: a low aggressiveness and a pronounced organotropism (the exclusive occurrence of pancreatic metastases).

### 8.1. Low Aggressiveness

The long interval to the onset of isPMRCC (~10 years); the favourable treatment results (75% 5-year survival rate); as well as the slow spontaneous course (42% 3-year survival rate [21], median survival 27 months [155]), which can be deduced from casuistic presentations as well as from single and multi-centre reports, are observations that reflect low aggressiveness of the tumour cell clones involved. This low aggressiveness leads to a late and, above all, very slow increase in the tumour burden—whether due to an increase in the number and organ site(s) of metastases or an increase in the volume of the individual metastases. Three mechanisms are involved in the development of this slow tumour growth.

#### 8.1.1. Genetic/Epigenetic Observations

The genome of the clear cell RCC was decoded in 2013 [299]. It is characterized by the absence or mutation of the Von Hippel-Lindau tumour suppressor gene located at 3p25, and the frequent inactivation of the chromatin-modifying genes PBRM1, BAP1, and SETD2 [266,300] located on the same 3p chromosomal region [301]. Investigations have reported that PAB1 mutations occur in approximately 10% of ccRCC while PBRM1 mutations have a prevalence of about 40% [301,302,303]. Carlo and coll. [303] demonstrated that the presence of PAB1 was associated with worse OS, whereas PBRM1 mutations seemed to correlate with a tendency toward superior OS [287]. In an investigation of Voss and coll. [304], any mutation of PAB1 was associated with reduced OS (*p* = 0.0008) as well as the absence of alterations of the PBRM1 gen *p* = 0.0035.

In 2018, the first studies were presented looking for genetic changes that control the metastasis behaviour of the RCC [305,306]. Turajlic [305] investigated the genetic changes relevant to the metastatic behaviour of RCC. Three genetic changes proved to be decisive for the metastasis potential of the RCC: (a) Two chromosomal changes were frequently found in the cell clones. The loss of 9p21.3 and the less pronounced loss of 14q31.2 are highly specific changes at the onset of the metastasis process; (b) the metastatic potential of clear cell RCC is reduced by low intratumoural heterogeneity and a small proportion of somatic copy-number alterations; (c) distinct patterns of metastasis are caused by punctual and branches evolution. In the course of this study—particularly interesting for the isPMRCC problem discussed here—three isPMRCC cases were also recorded and presented in detail. These PM showed a genetic profile different from the metastases in other organs: The isPMRCC were distinguished by the lack of 9p loss and a significantly lower weight genome instability index. Finally, in 2020, Singla and coll [244] conducted a comprehensive study on the genetic characteristics of pancreatic metastases that occur solitarily in the pancreas or in the context of multi-organ metastasizing RCC. (Of the 31 patients presented, 21 (68%) had metastases to additional organ site(s) and 10 patients (32%) had isolated PM). They found genetic changes in the PM cell clones, which are associated with less aggressive disease (e.g., low frequency of copy number alterations associated with aggressiveness and of 9p, 14q, and 4q loss; further a low frequency of BAP1 mutations that is associated with aggressive disease, and a high frequency of PBRM1 loss (>75%), which is associated with less aggressive disease [280,304,307,308,309,310]. Singla also observed a high sensitivity to TKI therapy, which correlated with the behaviour of biomarkers as angiogenetic markers were increased. An examination of the determinants of response to TKI therapy in mRCC was presented by Hakimi [309], who identified angiogenesis as one critical determinant of response to TKI therapy and concluded that the upregulation or downregulation of angiogenesis triggered by changes in PBRM1 and PAB1, respectively, provide an explanation for differences in the clinical behaviour associated with these mutations. Since the signature from loss of function mutations of PBRM1 and PAB1 is particularly pronounced in PM of RCC (PAB1 3%, BPRM1 77% [244]), this signature is able to explain the response to TKI treatment. Wang and coll. [311] examined the empirically-defined tumour microenvironment gene expression signatures of the RCC. This analysis allowed two subtypes of RCC to be identified: an inflammatory subtype (enriched for Treg, NK cells, neutrophils, macrophages, B-cells and CD8+ T cells) and a non-inflammatory subtype, which is characterized by enrichment for endothelial cells and low frequency of macrophages, B cells, T cells, NK cells, and neutrophils; BPRM1 gene loss; and increased angiogenesis associated with pancreatic organotropism [244,312].

In summary, these studies prove that the occurrence of PM in mRCC is associated with cell clones that have a lower aggressiveness and that can be distinguished from observations with extrapancreatic metastases by lack of loss of 9p, lower weight genome instability index, low frequency of BAP1 alterations, and a high frequency of PBRM 1 loss. Genetic studies thus provide a correlate for the clinical experience of the more favourable course of mRCC with pancreatic metastases than observations without PM [204,244,282,283,284,285,286,287]. However, these genetic and clinical studies are based on the analysis of groups that either exclusively or predominantly contained PM with additional multiple organ site(s) metastases. Whether these results can therefore be transferred without restriction to the small group of isPMRCC discussed here must remain unanswered at the moment, since a specific study on the isPMRCC has not been presented. The particularly favourable clinical course and the extreme organotropism suggest at least a special causative mechanism. On the one hand, therefore, the exceptional course of isPMRCC can only be the consequence of a simple increase in the effect of the genetic/epigenetic changes already detected in PM with additional distant metastases. However, it is also possible that additional—as yet unknown—genetic/epigenetic changes are active in isPMRCC. An answer to this question must therefore be left to future studies.

The observation that a subtype of a metastatic solid tumour—the isPMRCC—manifests itself by an extravagant organotropism as well as by a favourable clinical course, and that this independent behaviour has now been supported by genetic studies is extremely unusual. In this pronounced form, a similar peculiarity has not been demonstrated in other solid tumours. Even for adenocarcinoma of the pancreas (DAPA)—just to give a comparative example—decisive genetic/epigenetic characteristics have already been deciphered. ACPA is a cancer with a low overall tumour mutation burden, the majority of which is less than 50 mutations [313]. The genetic landscape of DAPA genomes is notable for four frequently mutated genes [313,314,315,316,317]. These include mutations of the oncogene KRAS (frequency >90% [313,314,315,316])—leading to uncontrolled activation of cell proliferation and survival pathways, of the tumour suppressor gene TP53 (70%)—permitting bypass of control checkpoints at the level of DNA damage, and (with lower frequency) of SMAD4—resulting in aberrant signalling by TGF-β mediating growth arrest and inducing apoptosis, and of CDKN2A—a negative regulator of G1 to S transition [314]. As these four genes contribute to pancreatic carcinogenesis, they are classified as driver genes for this tumour. These main driver mutations are mostly maintained both in primary tumours and in corresponding metastases [314,315,318]. Furthermore, gene mutations of MLL3, TGFβR2, ARID1A, and SF3B1, mutations in DNA-damage repair genes (ZIM2, MAP2K4, BRCA1-2) and in chromatin modification-involved genes (EPC1, ARID2) were observed [314,315,316]. As with other tumours, subtypes with prognostic and biological relevance can be distinguished on the basis of gene profiles [315]: Bailey [319]—squamous, immunogenic, pancreatic progenitor, aberrantly differentiated exocrine; Moffitt [320]—classical vs. basal like (11 vs. 19 mon., *p* = 0.007; and normal vs. activated stroma subtype *p* = 0.037); and Waddell [321]—stable, locally rearranged, scattered and unstable subtype). Furthermore, McIntyre [317] observed genomic alterations in KRAS and TP53, which are associated with worse outcomes and Yachida [318] reported that the number of altered driver genes is correlated with overall survival (*p* = 0,041) and disease-free survival (*p* = 0.008). Finally, loss of function mutation of suppressor gene SMAD4 is highly associated with increased propensity to metastasize and poor prognosis [314,322].

A subtype of ACPA, which can be separated analogously to isPMRCC by highly specific organotropism, very favourable prognosis, and specific genetic profile, is not reported so far in ACPA. On the contrary, exome sequencing of a cohort of eight very long-term survivors of ACPA found no differences in somatic mutations to explain the improved biology of tumours from these rare patients compared to the majority of patients with ACPA [323].

#### 8.1.2. Epigenetic Factors

The SSM postulated and suspected for the development of the isPMRCC is also suitable to contribute to the favourable course of the isPMRCC observations. As shown, there is evidence for addressing the exclusive growth of RCC metastases in the pancreas as a result of a highly selective and effective SSM, which allows circulating tumour cells to grow and mature to metastases only in the pancreas. However, if tumour cells outside the pancreas cannot survive—or, as is equally conceivable, are forced into a dormant, non-growth state for years [324,325]—an adequate therapy of pancreatic metastasis either leads to a definite tumour cure or, in the case of dormant cells, to a prolonged progression-free interval.

The extrapancreatic absence of viable metastatic tumour cells may also explain the lack of relevance of the risk factors mentioned at the beginning. These risk factors only describe the probability that after removal of the macroscopically detected PM, the presence of occult micrometastases in distant organs can be expected, from which tumour progression will occur later. However, if outside the pancreas no further occult micro-settlement can take place in isPMRCC, these risk factors must necessarily be negative.

#### 8.1.3. Genetic Stability of isPMRCC Clones

In addition to the low aggressiveness of the tumour cells, which manifest themselves in a limited tendency to metastasize and slow tumour growth, another characteristic of the cell clones is important, which is decisive for the favourable course: According to the data so far, the progressive dedifferentiation of the tumour cells with increasing tumour age is unusually low.

As the study of Turajlic shows [305], there are remarkably low genetic differences between the primary RCC and the three isolated PM occurring 15 years later, which underlines a high genetic stability of these tumour cell clones. Singla [244] reports a consistent result: tumours and metastases from patients with PM clustered together suggest limited evolutionary divergence. This certainly differs from the behaviour more common in malignant tumours, that as the tumour continues to develop (be it primum or metastasis), more and more undifferentiated and aggressive cell clones emerge and prevail, which ultimately determine the fatal clinical course.

The epidemiological data presented under recurrence (Section 5.2) further confirm this tumour cell stability. Even with tumour progression after isPMRCC therapy, only single organ metastasis occurs again in two out of three of the cases. Of these, about one in five even maintains the exclusive growth in the pancreas. The genetic stability of the isPMRCC cell clones and the resulting long absence of more aggressive cells is thus another specific feature of the isPMRCC, which is responsible for the favourable course. Which genetic and epigenetic changes and endowments are linked to this behaviour is still unknown to our knowledge.

### 8.2. Pronounced Organotropism (Exclusive Occurrence of Pancreatic Metastases)

The genetic changes discussed above are quite likely to provide a plausible explanation for the better prognosis of isPMRCC due to low aggressiveness and metastasis potential. However, the lower aggressiveness does not explain the exclusive growth of metastases in the pancreas. This unusual metastasis behaviour requires a highly selective selection mechanism, which allows the occurrence of metastases only in the pancreas and completely prevents extrapancreatic metastasis. The cell clones leading to the isPMRCC thus have two unusual characteristics: On the one hand, a low level of aggressiveness, the specific genetic causes of which have already been elucidated at least in part in recent years. On the other hand, these cell clones of the non-inflammatory mRCC subtype have also acquired properties that—by epigenetic mechanisms—trigger a highly specific SSM in possible host organs, which ultimately allows the settlement of metastases only in the pancreas. Biochemical studies on isPMRCC observations—whether genetic or epigenetic—that explain the exact mechanism that leads to the exclusive settlement of metastases in the pancreas have not been presented to our knowledge due to the rarity of this entity. Therefore, only analogical conclusions of more frequent and better studied tumour entities can be used as possible clues. Currently, at least five mechanisms can be defined that can trigger an organotropism [326] in metastasis settlement.

#### 8.2.1. Impact of microRNA (miRNA)

Based on the efficacy of an SSM, primary tumours that produce a large number of cells with different properties will gain an advantage in metastases formation, as this increases the chance that a suitable circulating tumour cell will reach a suitable host organ to mature for metastasis. The RCC is characterized by a great heterogeneity [299,300,312,327,328], for which a large number of miRNA with altered, disturbed expression behaviour is also responsible.

MiRNA are a group of small RNA (16–22 nucleotides) that control the expression of target genes. These miRNAs can also influence the metastasis process, e.g., by influencing epithelial-mesenchymal transformation, migration, and settlement [329,330,331,332,333,334,335,336,337,338,339,340,341]. Several investigators have shown the large number of altered miRNA in RCC [342,343,344,345]; furthermore, it has been shown that the miRNA profiles of metastatic and non-metastatic RCC differ [345,346,347] and that miRNA signatures can be used as a prognostic factor [343,344,346,348]. It was also shown that miRNA expression in distant metastases of the RCC differs depending on the metastasis location in the lung, bone, or brain [348]. These results thus show an interaction between miRNA profile and metastasis potential and metastasis localization. Whether a specific miRNA signature is responsible for isPMRCC currently remains unclear, since such a study has not been carried out due to the rarity of the isPMRCC.

#### 8.2.2. Pre-Metastatic Niche (pmN)

The pmN is the result of an epigenetic phenomen: the ability of tumours to influence a target organ long before metastases develop in such a way that they form a special microenvironment, a “fertile soil”, which enables subsequent metastasis formation by supporting the seeding, colonisation, survival, and outgrowth of the metastasizing tumour cells [349,350,351,352]. Involved in the formation of pmN are primary tumour-derived components (exosomes and microvesicles) [349,353,354,355,356,357], tumour mobilised bone marrow-derived cells [353], and the local microenvironment [355,358], which initiate and control this process in such a way that a special microenvironment—a “fertile soil”—is created for the circulating tumour cells, which enables the formation of metastases by inflammation, immunosuppression, increased angiogenesis, vascular leakiness, and extracellular matrix remodelling [352].

The development of pmN thus takes place in an ordered sequence of interactions of tumour cell and host organ properties. Since the profile of these special properties varies in both individual primary tumours and host organs, this inevitably leads to organotropism in metastasis development, which is a characteristic of the pmN [352,359]. For the RCC, its ability to form a pmN in the lung was already demonstrated in 2011 [358]. A pmN of the RCC in the pancreas has not (yet) been detected to the best of our knowledge.

#### 8.2.3. Immune-Surveillance

The cells of the primary tumour as well as circulating and metastasizing tumour cells are recognised and combated to varying degrees as foreign cells by the immune system. Thus, immunity is an effective defence against metastasis [360]. A fatal “counter-strategy” of individual malignant tumours is therefore their ability to “block” the immune response [271]. This realization led to the development of IT, which tries to reverse the elimination of the immune system. Blockade of the immune system also occurs in RCC, so that IT is generally effective in this tumour [274,275]. The importance of the immune defence in mRCC was already more anticipated than understood very early on in the extremely rare spontaneous remission of metastases—also in the pancreas [85]—which were attributed to a modified immune defence [361,362,363].

It is, therefore, all the more surprising that in the previously cited study by Singla [244], IT in PM of the RCC was not effective, whereas the treatment with TKI was highly effective. This suggests that the cells of this RCC entity are very well recognized by the body as foreign and combated, so that an additional IT remains ineffective. Of course, it remains unclear why only in the pancreas the immune defence is ineffective and thus triggers an organotropism.

The insensitivity to IT with simultaneous sensitivity to antiangiogenetic therapy with TKI could be explained in the above-mentioned study [244] by the behaviour of biomarkers: While angiogenetic markers were increased in the PM and the associated primary tumours, inflammatory markers remained low (e.g., enrichment for endothelial cells and low frequency of macrophages, B cells, T cells, natural killer cells, and neutrophils). This underlines that the occurrence of PM is linked to the non-inflammatory subtype of RCC [311], characterized by increased angiogenesis, which includes a lack of inflammatory component and thus a non-response to IT [244,312].

#### 8.2.4. Chemokine Receptor—Ligand Mechanism

A successful interaction of a chemokine receptor located at the tumour cell surface and a suitable ligand of the host organ is a necessary prerequisite for the activation of numerous signal transforming pathways, which are critical in the early metastatic process [364,365]. Since the chemokine endowment is specific to the tumour cell and the level of the specific ligand organ, a successful interaction can only take place in those tissues where the receptor and ligand match exactly. This must necessarily lead to an organotropism; e.g., breast cancer cells express high levels of CXCR4 and CCR7, which are responsible for the metastasis formation in LN, lung, liver, and KM, as these organs are rich in corresponding ligands CXCL12 and CCL21 [Chambers3471].

#### 8.2.5. The Metabolic Adaptation of Tumour Cells

In the stage of early avascular tumour growth, micrometastasis goes through a critical phase, as the supply of energy carriers by diffusion alone is critical. Each host organ presents the embolized tumour cells with the challenge of coping with the different energy carriers and oxygen or oxygen stress. Only those tumour cell clones that are able to use all energy carriers provided by the respective host organ through metabolic adaptation will remain successful [326,366,367,368,369,370,371]. This means that those tumour cells are preferred in metastasis settlement that can best use the energy source(s) available locally. The brain, for example, has the highest energy demand. Although glucose is the primary source of energy, the brain possesses the ability to adapt its metabolism when glucose is low and to metabolize acetate, ketone bodies, or short- and medium-chain fatty acids as alternative energy sources [367]. The liver environment on the other hand is notably more conducive for cells that display a high glycolytic activity and are adopted for low oxygen rate for example by using creatine metabolism, too [368]. It is therefore tempting to assume that those embolised cells will gain an advantage to colonise that are able to overcome metabolic barriers by metabolic plasticity, which enables them to use all resources available in an individual organ [366,369,370,372]. Here again, a successful interaction of the host organ (provision of energy carriers) and tumour cells (utilization of energy carriers) is a prerequisite—i.e., a SSM, which always triggers an organotropism.

Tumour-specific rapid cell proliferation is associated with an increase in cell metabolism, which in turn affects the microenvironment. On the one hand, poor perfusion/hypoxia-induced glycolysis leads to the release of lactate; and on the other hand, the oxidative energy metabolism leads to the release of CO_2_, which forms with H_2_O the volatile acid H_2_CO_3_. As a result of their increased growth, the tumour cells modify the microenvironment to acidic pH [373], which in turn gives an advantage to cell clones in the tumour that are resistant to acidic PH values but also influences the metastasis ability [374,375]. However, in the case of isPMRCC with their particularly protracted course, acidosis caused by rapid tumour growth cannot be of particular importance. On the contrary, the isolated growth of cells in the pancreas at least suggests the presence of cell clones that are well adapted to an alkaline environment. This would inevitably lead to an organotropism in the pancreas characterized by an alkaline environment, whereas in extrapancreatic organs, the formation of metastases is impeded or impossible.

##### Limitations

The limitations of the presented review with meta-analysis are the retrospective nature of the analysed casuistic reports, the long period of investigation, and the possibility that a bias in the compiled papers cannot be excluded. However, this methodological limitation is partially compensated by the confirmatory results of current large single and multicentre analyses.

## 9. Conclusions

isPMRCC is a tumour entity with a unique disease biology [216] that triggers an unusually low aggressiveness, with aresulting protracted course and favourable treatment outcomes. The genetic changes that are responsible for the low aggressiveness are at least partly explained by research in recent years. However, two peculiarities of isPMRCC are not elucidated in their genetic/epigenetic causes: On the one hand, the high genetic cell stability, and on the other hand, the particularly pronounced SSM, which is the basis for the isolated occurrence of PM. Therefore, biochemical studies seem useful to elucidate this highly selective metastasis process. This is all the more so because the largely uniform clinical course of all observations suggests that the phenomenon is due to an equally uniform pathomechanism that remains constant over a long period of time. This clarification could lead to a better knowledge and understanding of the processes involved in metastasis settlement and growth, which in turn is a prerequisite for the development of therapeutic concepts to slow down or completely block the metastasis process.

## Figures and Tables

**Table 1:** Analysis of 1433 isolated pancreatic metastases from RCC (N = number of cases with adequate documentation).

Variable	Data	%
**Casuistic case reports**	573	
**Renal cell carcinoma**		
EXSide affected: left:right:bil. (N = 476)	253:216:7	53.1:45.4:1.5
TNM category: T (N = 87)	T1 13; T2 35, T3 37, T4 2	12.8, 40.2, 42.5, 2.3
TNM category: N (N = 79)	N0 73; N1 6	92.8; 7.2
TNM category: M (N = 68)	M0 67; M1 1	97.2; 2.8
Histology clear cell (N = 420)	411	97.8
**Pancreatic metastases**		
Age at diagnosis (N = 833) EX	63.7 (SD 9.9)	
Synchron.-metachron. (N = 839)	62:777	7.4:92.6
Interval to metastasis (N = 855)	9.6 (SD 6.5)	
Solitary/multiple (N = 733) EX	451; 282	63.6; 36.4
Number of multiple metastases (N = 115)	3.1 (SD 1.5)	
Site: head; corpus; cauda; multiple (N = 419)	141; 67; 85; 129	33.4; 15.9; 20.1; 30.6
Size (mm), (N = 417)	32.6 (SD 20.5)	
LN metastasis (N = 517)	32	6.2
**Outcome of surgery**		
Cum. 5-year survival rate (N = 432)		75.7
Recurrent disease (N = 301)	125	41.5

**Table 2:** Correlation between side of renal cell carcinoma and site of metastasis within the pancreas (N = 188; *p* = 0.876).

Localisation of isPMRCC		Side of RCC	
	Left	Right	Bilateral
Head	51	43	2
Body	23	24	1
Tail	26	17	1
Total	100	84	4

**Table 3:** Early metastatic cascade.

Step	Characteristics
Premetastatic niche	Inflammation, immunosuppression, organotropism, ECM-reprogramming, angiogenesis, vascular permeability
Intravasation	Proteases, signalling molecules: TGFβ, EGF-receptor
Transport	Mechanical stress, interaction with blood components
Docking	Mechanical, selectin & integrin binding
Extravasion	EMT, mesenchymal/amoeboid transition
Colonisation	Dormancy, vascular network formation, immune response

**Table 4:** Recurrent disease following treatment of isPMRCC.

Author	Year	N	Median Follow Up (Mon)	Median Time to Recurrence (Mon)	N and % Recurrence	N and % Single Organ Metastases	N and % Pancreatic Metastases
Blanco-Fernandez [247]	2021	116	43	33	62/116 53	57/62 92	17/62 27
Malleo [249]	2021	69	141		48/67 72	30/48 61	13/48 27
Di Franco [238]	2020	21	77		9/21 43		
Fahlbusch [239]	2020	12	48		1/12 8		
Milanetto [241]	2020	31	68	25	16/31 52		5/19 25
Chikhladze [18]	2020	20	76	14	13/20 65		
Anderson [8]	2020	29			16/20 55		
Ma [232]	2019	13	26		0/13 0		
Chatzizacharias [213]	2017	13		28	5/13 38		
Yagi [4]	2017	7			3/7 43		
Dong [207]	2016	5			4/5 80		
Fikatas [208]	2016	18	49		7/18 39		1/7 14
Benhaim [198]	2015	20	69		11/20 55		1/11 9
Moletta [194]	2014	9	56	40	4/9 44		2/4 50
Schwarz [195]	2014	62	91	26	37/62 60	26/37 70	9/37 24
Niess [9]	2013	16			7/16 44		
Yazbek [179]	2012	11			6/11 45		5/11 45
You [174]	2011	7			2/7 29		
Law [124]	2009	14	32		8/14 57		5/8 62
Total					259/482 53		

**Table 5:** Influence of PM on OS (months) in mRCC with multi-organ sites and single organ site metastases.

	**RCC with Multiple Organ Metastases**		**RCC with Single Organ Metastases**	
	Without PM [204,244,282,283,284,285,286,287]	With PM [204,244,282,283,284,285,286,287]	Isol. PM [6,11,18,106,152,159,174,179,189,197,208,213,238,241,246]	Isol. Lung Meta. [288,289,290,291,292,293,294,295,296,297,298]
OS min.–max.	18–35	29–101	48–148	30–94
Median OS	23	41.7	75	56.2
